# Supplementary material for: The Right to Development and disparities in healthcare access: Qualitative study from rural Ontario, Canada
Source: PLOS Glob Public Health. 2026 Jun 1;6(6):e0005758. doi: 10.1371/journal.pgph.0005758 (PMC13225652; doi:10.1371/journal.pgph.0005758)
Supplement: Attachment — Submitted filename: Response to Reviewer - POLS Public Health.docx [file pgph.0005758.s003.docx]

**Response to Reviewer**

**Response to author according to page indicated in the PDF**

**Title page 1**

**The reviewer said regarding :**These authors contributed equally to this work, “From what's written in the paper, this doesn't seem to be the case. No information is provided as to the roles played by the other three co-authors. Rather, the manuscript strongly suggests that all of the work was analysed and written by the first author. Please clarify the roles played by the other authors.”

**Response**: Thank you for this comment. We apologize that the roles of the co-authors were not sufficiently clear in the original manuscript.

“The first author (doctoral student) initiated the project from its inception and was responsible for the overall study design, conducting the interviews, data analysis, and drafting the manuscript. The co-authors contributed to the study through supervisory and collaborative roles. Specifically, they provided administrative and logistical support, contributed to the conceptual development of the project, offered methodological guidance, and gave ongoing constructive feedback throughout the data analysis and writing process. All co-authors critically reviewed and revised the manuscript and approved the final version for submission.”

1. **Reviewer comment in page 4“**State in full in first mention” for RtD

**Response:** Right to Development

2. **Reviewer comment in pages 4-5 said:** “Perhaps this sentence could be combined with the previous one -- "While RtD discourse often focuses... in New Zealand, and to address regional inequalities in the European Union". Otherwise, this sentence seems oddly placed.”

**Response:** While Right to Development (RtD) discourse often focuses on the Global South, it has also been applied in developed contexts, including Indigenous participation in resource management in New Zealand [27] and efforts to address regional inequalities in the European Union [24].

**3. Reviewer comment in page 7 said:** This is a welcome critique of liberal theories, although the conclusion sentence between lines 156-158 seems incomplete. Did you mean to contrast Sen and Nussbaum from other utilitarian/basic needs/iberal theories? If so, it's not clear how they differ (since guaranteeing essential groups and securing minimum living standards doesn't quite equate to focusing on "only economic indicators").

**Response:** This limitation highlights a key critique of liberal theories, such as Dworkin’s equality of resources, which protect individuals from bad luck without directly addressing accumulated advantage [37]. In contrast, Sen’s and Nussbaum’s capabilities approaches move beyond utilitarian and basic-needs frameworks by focusing on securing substantive freedoms and essential capabilities, such as the ability to access healthcare, education, and political participation, necessary for individuals and groups to lead dignified lives.

**4. Reviewer comment in page 7 said:** “The pending Convention on the RtD may strengthen accountability, but within the constraints of the current UN system.” Briefly, what are these constraints?

**Response:** Thank you for this comment. I have clarified the reference to “constraints” by briefly specifying structural limitations of the current UN system, including reliance on state consent, political will, and limited enforcement mechanisms. I said: The pending Convention on the Right to Development may strengthen accountability, but its impact will remain constrained by structural limitations of the current UN system, including the non-binding nature of many enforcement mechanisms, reliance on state consent and political will, and limited capacity for compulsory compliance.

4. **Reviewer comment in page 8 said:** For the sentence of “ Federal agencies such as the Department of Canadian Heritage and Citizenship and Immigration Canada continue to support these principles.” Unclear... did you mean the Department of Canadian Heritage, and Immigration, Refugees and Citizenship Canada? If so, I'd suggest rewriting to "Federal agencies such as the **Department of Canadian Heritage, and Immigration, Refugees and Citizenship Canada, continue to support these principles**".

**Response:** I have revised the sentence to improve clarity.

Federal agencies such as the Department of Canadian Heritage, and Immigration, Refugees and Citizenship Canada, continue to support these principles.

5. **Reviewer comment in page 9 said:** For the sentence of: Guided by inductive reasoning, the study’s qualitative research design was developed iteratively, with interview questions refined as new themes and participant perspectives emerged. Said: Braun and Clarke -- which the authors claim to have used -- have, among other things, repeatedly emphasised that "themes do not emerge". I would strongly suggest reading the following article, which highlights important points to note when using thematic analysis: Morriss, L. (2024). Themes do not emerge. An editor’s reflections on the use of Braun and Clarke’s thematic analysis. Qualitative Social Work, 23(5), 745-749. You might also wish to consider the following: Braun V, Clarke V. A critical review of the reporting of reflexive thematic analysis in Health Promotion International. Health Promot Int. 2024 Jun 1;39(3):daae049. doi: 10.1093/heapro/daae049

**Response:** Thank you for this important methodological clarification. I have revised the language to align with Braun and Clarke’s reflexive thematic analysis approach, avoiding the implication that themes “emerge” independently of the researcher. The revised text now reflects the active and interpretive role of the researcher in theme development, consistent with Braun and Clarke’s work and recent methodological critiques. We appreciate the suggested references, which informed this revision. “Guided by inductive reasoning, the study’s qualitative research design was developed iteratively, with interview questions refined as the researcher actively identified and constructed themes through ongoing engagement with the data and participant perspectives.”

6. **Reviewer said in page 10:** “Please provide a reference that supports these claims (I do not disagree with what you have written here, but supporting your assertions for references would strengthen your claim re. the appropriateness of research methods). For the sentence of : To ensure a diverse and information-rich sample, both purposive and snowball sampling methods were used. Purposive sampling enabled the intentional selection of individuals with direct experience and knowledge relevant to the research questions, while snowball sampling facilitated additional recruitment through participant referrals, particularly useful for reaching harder-to-access groups.

**Response**: [54]. Vincent, B. W. (2018). Studying trans: Recommendations for ethical recruitment and collaboration with transgender participants in academic research. Psychology & Sexuality, 9(2), 102-116.

7. **Reviewer said in page 11:** “I wonder if you could say "the first author, a doctoral student (include other traits that reveal positionality, e.g., of recent migrant background, etc) conducted all interviews. At the start of each Zoom interview, the interviewer...." for this statement: At the start of each Zoom interview, the student investigator reviewed the ICF with the participant and invited questions to confirm understanding. Verbal informed consent was obtained before the interview began and was documented by the student investigator on the study consent record (including the investigator’s name, signature, and date).

**Response:** The first author, a doctoral student, conducted all interviews. At the start of each Zoom interview, the interviewer reviewed the informed consent form (ICF) with participants and invited questions to confirm understanding. Verbal informed consent was obtained prior to the interview and documented in writing by the interviewer on the study consent record, including the interviewer’s name, signature, and date.

8. **Reviewer page 10 said:** “Interviews were digitally recorded via Zoom, and recordings, transcripts, and study documents (including consent documentation) were stored on an institutionally secure server with restricted access”.

**Response:** Interviews were digitally recorded via Zoom, and recordings, transcripts, and study documents (including consent documentation) were stored on an institutionally secure server with access restricted to the investigators (the first author and the principal investigator), in accordance with the approved institutional ethics protocol.

9. **The Reviewer said in page 10:**

“The inclusion of this table does not contribute to your argument or readers' understanding of the manuscript in any way. I'd suggest removing it.”

**Response:** The table removed.

10. **Reviewer said on page 11:** “How were themes determined?

Response: Thank you for this comment. We appreciate the opportunity to clarify this point.

Although the interview guide was informed by existing literature on healthcare access barriers, the data analysis followed an inductive approach. The themes were not predetermined. Instead, they emerged directly from the interview data through an iterative process of coding and comparison. After transcription, the first author conducted open coding of the interviews, allowing patterns and themes to develop from participants’ accounts. These themes were subsequently refined through discussion with the co-authors to ensure analytical rigor and coherence.

11. **Reviewer said on page 10-11** for the statement of “The interviews were conducted over Zoom, with both audio and video features turned on and off to ensure participant privacy and minimise the risk of internet disruptions.” Confusing phraseology - did you mean you only used the audio functionality, with video turned off?

**Response:** We apologize for the confusing phrasing.

All interviews were conducted via Zoom using audio recording. Use of the video function was optional and determined by participant preference; some participants chose to enable video, while others preferred to keep it turned off. This approach was adopted to respect participant comfort and privacy while ensuring interview continuity in cases of internet instability.

12. **Reviewer said on page 11,** regarding the sentence of: Each interview was scheduled to last up to 60 minutes. He stated that: How long did the interviews take on average?

**Response:** Each interview was scheduled for up to 60 minutes; the average duration was approximately 45 minutes, with interviews ranging from **[minimum] to [maximum] minutes.**

13. **The reviewer said on page 11 for the statement of :** As a token of appreciation, participants received a 20–25 CAD e-transfer, with the amount reflecting the level of effort and participation during the interview. “How was this determined? Do you have any references that would support this tiered approach to participant compensation? Otherwise it raises some ethical questions.”

**Response:** Thank you for raising this important ethical consideration.

Participant compensation was not based on the quality of responses, content of the interview, or any form of performance evaluation. Rather, the modest variation in compensation (20–25 CAD) reflected practical considerations such as interview length and scheduling burden. All participants were informed in advance of the compensation range, participation was entirely voluntary, and compensation was provided regardless of whether participants completed the full interview. The compensation amount and procedure were reviewed and approved by the institutional research ethics board as part of the overall study protocol, prior to data collection. Individual interviews were not reviewed separately; rather, the approved protocol governed all interviews conducted in the study.

14. **Reviewer said on page 11** regarding the statement of: The process began with deep engagement with the transcripts, reading them multiple times and creating reflexive notes to capture early insights and personal assumptions. “Who read the transcripts - only the first author?

**Response:** The transcripts were read and initially analyzed by the first author. This involved repeated reading of the transcripts, reflexive note-taking, and the development of preliminary codes and themes. The emerging analysis was subsequently discussed with the co-authors, who provided critical feedback and guidance to support reflexivity, analytical rigor, and interpretation.

15. **Reviewer said on page 11** on the statement of by whom: Coding was then **carried out** inductively and interpretively, focusing on generating analytic observations of participants’ experiences of rural healthcare rather than relying on predetermined or line-by-line codes. “ By whom”?

**Response:** The inductive and interpretive coding was conducted by the first author. This involved systematically coding the transcripts to generate analytic observations of participants’ experiences. The coding framework and emerging interpretations were subsequently discussed with the co-author (the principal investigator), who provided critical feedback and contributed to the refinement of themes and interpretations.

16. **The reviewer said on page 11** regarding the statement of: These early themes were then reviewed across the whole dataset, refined, merged, or removed to ensure each theme had a clear central concept. “Reviewed by whom?

**Response:** The review and refinement of early themes were conducted by the student investigator (first author). This process involved revisiting the entire dataset to refine, merge, or remove themes to ensure that each had a clear and coherent central concept. The refined themes were subsequently discussed with the co-author (the principal investigator), who provided constructive feedback and contributed to the final interpretation.

17. **The reviewer said on page 11** regarding the statement of : Consistent with RTA, themes were viewed as interpretive accounts shaped through the researcher’s engagement with the data, not as objective findings that simply “appeared.” Yes, this is closer to B&C's TA approach, although the word "emerged" appears earlier in the manuscript.

**Response:** We agree that our analytic approach aligns with Braun and Clarke’s reflexive thematic analysis, in which themes are understood as interpretive constructions shaped through the researcher’s active engagement with the data. We acknowledge that the earlier use of the term “emerged” was inconsistent with this position. We have revised the manuscript to replace this wording and ensure conceptual and terminological consistency throughout.

18. **The reviewer said on page 11** regarding the statement of: To enhance transferability, the analysis and reporting incorporated detailed descriptions of the study context, rural Oxford County as a geographically spread, agricultural region influenced by winter conditions and significant travel distances, as well as participants’ social positions and healthcare experiences [56,57]. “For a journal with a diverse readership, it would be helpful to define terms that might be unfamiliar to those not as familiar with qualitative research. Would you be able to give a brief definition of transferability? What did Braun and Clarke say about transferability (if they said anything at all? This seems more in line with Guba & Lincoln...

**Response:** Thank you for this helpful comment. We agree that the term transferability may not be familiar to all readers and that clearer conceptual grounding is needed.

To enhance transferability that is, the extent to which qualitative findings may be applicable or relevant to other contexts through rich, detailed descriptions that enable readers to make their own judgments rather than relying on statistical generalization the analysis and reporting incorporated detailed descriptions of the study context. This included characterizing rural Oxford County as a geographically dispersed, agricultural region shaped by winter conditions and significant travel distances, as well as detailing participants’ social positions and healthcare experiences [56,57]. Transferability is a concept originating from Guba and Lincoln’s framework for establishing trustworthiness in qualitative research.

19. **Reviewer said on page 12** regarding the sentence of: Ethnic face barriers, such as ethno-racial stratification, social isolation and social support deficits. “Unclear/incomplete sentence?

**Response:** The analysis identified themes reflecting barriers faced by ethnic minority participants. These included **constraints in social support**, with sub-themes of **ethnoracial stratification, social isolation**, and **limited social support**, as well as themes related to **equity and inclusion in policy,** including **accessibility**, **resource allocation,** and **inclusive care.**

20. **The Reviewer said on page 13:** What's Woodstock? I would suggest perhaps using just one or two quotations rather than providing five of them. You could perhaps remove EM015 as it doesn't tell us anything (what is the consequence of the nursing staff being predominantly white? Why did the participant think that was a problem?), and retain EM025 and EM012 as your main point here seems to be ethnic segregation and how that relates to barriers to healthcare (a point you should analyse much more deeply rather than the cursory manner in which it is done here).

**Response:** Thank you for this detailed and constructive feedback. We agree that the manuscript initially included an excessive number of quotations and that greater analytic focus was needed.

EM025 adds: “I noticed that white people are just very separated from the other communities. They do not really, you know, mix in, or they just treat you as a brown person…They are not very friendly; they are just very separate.”

EM012 adds: “I feel like there are obviously some barriers related to healthcare, and like people of colour.”

These accounts highlight ethnic segregation as more than a background feature of community life; participants described it as a social context that shaped how they anticipated and experienced healthcare. EM025’s description of being treated “as a brown person” indicates a racialized sense of being marked as an outsider, which can produce heightened vigilance and uncertainty when interacting with institutions perceived as predominantly White. In this context, healthcare is not approached as a neutral service but as a setting where unequal treatment is possible and where belonging is not assumed. EM012’s comment that barriers exist for “people of colour” reinforces that participants understood these challenges as patterned rather than individual. These narratives suggest that social separation and limited intergroup contact may restrict access to informal support (e.g., guidance on where to seek care, how to navigate services) while also undermining trust and comfort in healthcare encounters. This can contribute to delayed care-seeking, reduced willingness to ask questions or advocate for needs, and diminished continuity of care mechanisms through which community-level segregation becomes a practical barrier to equitable healthcare access.

21. **The reviewer said on page 14:**

EM007 said: “From the so, the average they are treated Muslims differently. I do not know why?”

Such experiences point to the complex intersection of racial, cultural, identity and religious barriers that minority groups face. “The meaning of this part of the quotation is unclear.”

**Response:** This account reflects participants’ experiences of perceived religious discrimination that was felt but not always easily articulated or attributed to a single cause. EM007’s uncertainty “I do not know why” highlights how differential treatment may be experienced as subtle, normalized, or difficult to name, particularly when it intersects with racialized and cultural identities. Such ambiguity can make discrimination harder to challenge or report, while still shaping feelings of exclusion and vulnerability in healthcare settings. This illustrates how intersecting racial, cultural, and religious identities can compound barriers to equitable healthcare access, even when discriminatory practices are not overt or explicitly acknowledged.

22. **The reviewer on page 14 said regarding the sentence of:** Segregation and stigma result in minority individuals feeling isolated, fearful of judgment, and reluctant to seek help. “Minority ethnic individuals? "Minority individuals" is a vague term.

**Response:** Thank you for this comment. We agree that the term “minority individuals” was too vague. We have revised the manuscript to use more precise language and to explicitly refer to ethnic minority participants, consistent with the focus of the study.

Segregation and stigma contributed to ethnic minority participants feeling isolated, fearful of judgment, and reluctant to seek healthcare. In this study, ethnic minority participants refer to individuals who self-identified as belonging to racialized, ethnocultural, and/or religious groups that are minoritized within the local population context.

23. **Reviewer said on page 14 regarding the quote:**

EM019 said: “If I say Hi to them all morning, they will just ignore me… I think I am the only Black. If I am not mistaken.”

**“**Who is the subject of this sentence? Healthcare workers?

**Response:** EM019 described feeling ignored by co-workers in a factory workplace, stating: “If I say hi to them all morning, they will just ignore me… I think I am the only Black [person there], if I am not mistaken.” This quote illustrates everyday racialized exclusion that contributed to feelings of isolation and shaped expectations of exclusion in healthcare settings.

24. **Reviewer on page 14 said: check the sentence flaw.**

**Response:** EM001 also described how nonverbal cues conveyed disapproval more powerfully than words, stating: “Some people were very quiet and did not say anything, but their facial expressions suggested how they were feeling.”

**Reviewer said on page 14 regarding the “These narratives…”.** I have revised the manuscript to provide clearer contextual information when introducing participant quotations (e.g., participants’ family roles, migration background, or social positioning where relevant). We have also restructured this section to more clearly link quotations together within a coherent narrative, rather than presenting them as standalone excerpts. Specifically, I clarified how experiences of social isolation extended beyond individual adults to affect family members, including children, and strengthened the accompanying analysis to explicate these connections.

These revisions were made to enhance narrative logic, improve interpretive depth, and ensure that quotations are clearly situated within participants’ lived experiences rather than appearing as isolated statements.

25. **Reviewer said on page 14 regarding the sentence of:** Participants also highlighted broader social changes that have transformed the fabric of community life. “What are some of these "broader social changes"? Have they given examples?

**Response:** Participants did provide specific examples of changes affecting community life, including increased cultural diversity, shifts in community cohesion, and changing social norms that were perceived as contributing to social fragmentation and reduced interpersonal interaction.

26. **Reviewer said on page 15 regarding the quote of:** EM004 stated: “And part of this is also a breakdown of the social structure. There is no family structure anymore in most places in Canada. Parents are living on their grand…. So that's a social structure, deterioration problem as well.”

- The meaning of this quotation is unclear
- This is an important point . Is this participant saying that it's an issue among migrant communities as well, which makes it difficult to find support even within their own communities? Or, if you're referring to the lack of family structure within white Canadian communities, it might be helpful to be much clearer about why that would present a problem with accessing health care (as the connection between the two isn't immediately apparent).

**Response:** The participant was referring to a perceived erosion of family and community support structures in the broader Canadian context, rather than exclusively within migrant communities. The participant described this as a broader social change that reduced the availability of informal support networks, which they viewed as essential for navigating everyday challenges, including accessing healthcare.

27. **Reviewer said on page 16** regarding the sentence of: Analyse or expand on the implications, including the challenges faced by hospital staff, the potential for burnout, and the need for integrated social services in rural areas.

**Response:** This account points to systemic resource constraints within the education system that extend beyond schooling and shape healthcare access for children with disabilities and their families. Insufficient staffing, inadequate funding, and increasing class sizes place significant strain on teachers and support staff, limiting their capacity to respond to complex needs and increasing the risk of burnout. For families, these constraints contribute to fragmented care pathways, as schools often serve as key sites for identifying needs, coordinating services, and facilitating referrals to health and social supports. In rural contexts, where services are already limited, the lack of integration between educational, healthcare, and social systems further exacerbates delays in assessment and care. Participants’ accounts thus underscore the need for coordinated, cross-sector approaches that better support both service providers and families navigating multiple systems.

28. **Reviewer on page 17 said regarding the quote of:**

EM007 said: “Health is related to development. Yes, because we live in a developed country, right? So, when we were coming to Canada, I thought the medical system was perfect. But when I came here, I saw, oh my God! There is **a terrible situation**. Of course, these are free. Of course, these are free, but if anything is free, you know, we cannot compromise on the quality.” Did the participant say what about it was terrible? Otherwise, it would be very difficult to identify specific points of improvement.

**Response**: EM007 contrasted expectations of a high-quality healthcare system with their experiences of long wait times, delayed access to services, and perceived compromises in care quality, stating: “Health is related to development. Yes, because we live in a developed country, right? So, when we were coming to Canada, I thought the medical system was perfect. But when I came here, I saw, oh my God! There is **a terrible situation**. Of course, these are free. Of course, these are free, but if anything is free, you know, we cannot compromise on the quality.”

29. **The reviewer said about this quote on page 19:** EM023...clearly expressed this concern: “Please increase the standard of education of your doctors before you place them in the emergency room (ERs). ER is a very specialised branch and should be better than general practitioners. Doctors should be more qualified than a GP.” I'd suggest spelling out more clearly how this quote relates to your main point. (How does standard of education relate to inclusivity? The point this participant seems to be making is that they are worried that ER doctors aren't more qualified than GPs... it reveals perhaps their belief that ER doctors are higher on the hierarchy than GPs...) w

**Response:** The quote does not merely express concern about training, but also reveals assumptions about professional status and legitimacy. Suggesting that ER doctors should be “more qualified than a GP,” the participant implies a hierarchical distinction between emergency medicine and general practice, positioning the former as superior. Such assumptions implicitly devalue general practitioners and frame them as insufficiently competent for inclusion in emergency care settings. In this way, appeals to “standards” of education function as barriers to inclusivity by reinforcing exclusionary professional boundaries and undermining multidisciplinary practice.

30. **Reviewer said on page 19:** EM008 emphasised: “More work can be done to have spiritual support. For instance, in my case, someone who understands Islam can be close to them. The majority of the doctors do not really have any theological beliefs at all.” Who is the subject of this sentence? (I.e., to whom does "them" refer?)

**Response:** In this quote, “them” refers to patients. We have revised the text to clarify this explicitly by specifying “patients” in the quote and/or in the accompanying narrative to avoid any confusion. EM008 emphasised: “More work can be done to have spiritual support. For instance, in my case, someone who understands Islam can be close to them [patients]. The majority of the doctors do not really have any theological beliefs at all.”

31. Reviewer said on page 19: Together, **these recommendations demonstrate that achieving equitable healthcare requires comprehensive reforms.** Improving accessibility, ensuring fair resource allocation, and embedding inclusive, culturally sensitive practices are essential steps toward a system where everyone, regardless of background or location, can access high-quality care and feel respected throughout their healthcare journey.

Response: Together, these recommendations respond to identified gaps in equity by outlining comprehensive reforms aimed at improving accessibility, ensuring fair resource allocation, and embedding inclusive, culturally sensitive practices to support high-quality, respectful care for all.

32. **Reviewer said on page 20:** The reviewer said: Philosophers like Locke and Voltaire helped naturalise racial hierarchies [51], enabling dehumanisation and discrimination [62]. Said’s Orientalism illustrates how Western depictions of Arab and Muslim societies justified dominance [63], and these misrepresentations continue to influence policy and public attitudes. Addressing these long-standing boundaries requires more than policy change; it demands engagement with deeper cultural and social divides. said about the above statement: How might any of this be related to the topic at hand, in the Canadian context?

**Response:** I have revised the text to clarify how these historical ideas continue to shape contemporary Canadian institutions.

Philosophers such as Locke and Voltaire contributed to the naturalisation of racial hierarchies, enabling forms of dehumanisation and discrimination that have influenced Western institutions more broadly [51]. In the Canadian context, these intellectual traditions have shaped colonial governance, immigration policy, and institutional norms, including within healthcare systems [62]. Similarly, Said’s concept of Orientalism helps explain how enduring Western representations of Arab and Muslim societies continue to inform public discourse, policy debates, and professional assumptions in Canada [63]. These legacies are relevant to the present study, as they provide important context for understanding how racialised and Muslim patients may experience exclusion, stereotyping, or diminished legitimacy within healthcare settings. Addressing such inequities therefore requires not only policy change, but engagement with the deeper cultural and social boundaries that persist within Canadian society.

33. **Reviewer said on page 20**: Historical traditions shape ethnic identity formation, and Kallen proposes that present-day treatment by outsiders is discussed in Satzewich & Liodakis [51,64]. Unclear sentence (reorganise structure).

Response: Building on this regional distinction, Kilgour **[63]** argues that historical traditions play an important role in shaping ethnic identity formation, while **Satzewich and Liodakis** emphasise that present-day treatment by outsiders further influences how ethnic groups understand their social position and belonging [51,64].

34. Reviewer said on page 25: Western societies have historically placed themselves at the top of moral hierarchies, devaluing non-Western cultures [65, 66]. Did participants explicitly say any of this? Or is this your conclusion based on your data analyis?

Response: While participants did not state this explicitly, their accounts can be understood, when situated within existing scholarship, as reflecting broader historical patterns in which Western societies have positioned themselves as morally superior to non-Western cultures [65,66].

35. **Reviewer said on page 23:** Please include the scholar's name with the reference, for the sentence of cultural characteristics can also shape what [96] calls “monopolistic closure,” a process in which dominant groups use shared traits to secure exclusive access to valued resources, thereby excluding others

**Response:** Drawing on **Max Weber’s** analysis of social action and authority, cultural characteristics can also shape what [96] calls “monopolistic closure,” a process in which dominant groups use shared traits to secure exclusive access to valued resources, thereby excluding others.

36. **Reviewer said on page 25:** These insights align with my perspective that meaningful inclusion requires dismantling systemic barriers rather than relying on symbolic diversity measures. In my view, inclusive development depends on both removing obstacles and creating genuine avenues for participation among disadvantaged groups. Inclusion is essential in health policy, yet it cannot alone determine how resources are allocated. Who is the "my" that is referred to here -- the first author? Multiple authors are credited on this paper, so please clarify how the other authors were involved in the manuscript.

Response: These insights align with the perspective of the first author, a doctoral candidate, that meaningful inclusion requires dismantling systemic barriers rather than relying on symbolic diversity measures. From this perspective, inclusive development depends on both removing obstacles and creating genuine avenues for participation among disadvantaged groups. While inclusion is essential in health policy, it alone cannot determine how resources are allocated.

37. **The reviewer said on page 25**: As an Arabic minority researcher not residing in the rural community studied, my background informed my approach and sensitivity to equity issues, but required careful reflexivity to avoid bias. I would suggest being a bit more strategic with how you reveal your positionality. I would highly suggest reading Braun, V., Clarke, V., Hayfield, N., Davey, L., Jenkinson, E. (2022). Doing Reflexive Thematic Analysis. In: Bager-Charleson, S., McBeath, A. (eds) Supporting Research in Counselling and Psychotherapy, pp. 19-38 . Palgrave Macmillan, Cham. <https://doi.org/10.1007/978-3-031-13942-0_2>.

**Response:** The first author’s social positioning, including being an Arabic minority researcher and not residing in the rural community studied, shaped the interpretive lens brought to the analysis. In line with principles of reflexive thematic analysis, researcher subjectivity was understood as an inevitable and productive element of knowledge production rather than a source of bias to be eliminated. Reflexivity was therefore treated as an ongoing, analytic practice, involving continuous engagement with how assumptions, experiences, and interpretive decisions informed coding, theme development, and meaning-making throughout the analytic process. This approach emphasised transparency and critical self-awareness in the construction of themes, rather than positioning positionality as a fixed characteristic disclosed only at the outset of the research.

38. **The reviewer said on page 25 regarding Statement of:** The Oregon Experiment illustrates that while public input and transparency matter in healthcare rationing, fairness depends on the genuine inclusion of disadvantaged groups [104]. What is the Oregon Experiment? For a journal with as broad a readership as this one, ensure that terms are defined so that readers from various disciplinary and country contexts can understand your (important) work.

**Response:** The Oregon Experiment, a landmark U.S. healthcare rationing initiative from the late 1980s that used public consultation to rank and prioritise medical services for public funding, illustrates that while transparency and public input are important, fairness ultimately depends on the meaningful inclusion of disadvantaged groups [104].

39. **The reviewer said on page 26:** I'd suggest linking back to the recent RtD convention, which you mentioned in the introduction, and note how your study contributes to increasing interest in the field (I assume you're trying to support the importance of RtD and its potential?)

**Response:** This study explores healthcare access barriers faced by ethnic minority populations in rural Oxford County, Ontario, through the lens of the RtD. As highlighted in the recent RtD convention discussed in the introduction, there is growing international recognition that development must be participatory, inclusive, and grounded in human rights rather than solely in economic or institutional progress. The findings of this study contribute to this emerging discourse by illustrating how, in practice, limited social support and culturally insensitive care can prevent rural ethnic minority populations from fully participating in and benefiting from health and social systems, even where formal rights and services exist.

While inclusion is widely promoted as a core value within contemporary policy frameworks, the findings underscore that inclusion alone is insufficient. Policies must also guarantee universality, ensuring equal claims to services, and address practical accessibility by tackling the real-world barriers that persist despite formal entitlements. This aligns with the “basic needs” approach to development, which emerged from recognition that economic growth alone cannot eliminate poverty or secure well-being, and echoes Rawls’ concept of primary social goods. Both perspectives emphasise that just societies and effective development policies must prioritise the fundamental needs of the most disadvantaged, rather than focusing solely on aggregate progress or symbolic participation.

Accordingly, this study suggests that healthcare reform in rural Ontario must integrate procedural values central to the RtD, such as meaningful participation with substantive commitments to universality and accessibility. Addressing intersecting barriers requires moving beyond top-down policy design toward collaborative action, ensuring that individuals not only hold the right to healthcare but also possess the practical ability to realise that right. In this way, the study supports the growing interest reflected in the RtD convention by demonstrating the framework’s relevance to rural health equity in high-income countries. Advancing health equity is therefore both a matter of social justice and a necessary condition for sustainable development, requiring coordinated policy action, cross-sector collaboration, and accountability to those most affected by exclusion.

40. **Reviewer said on page 26.** “My thanks go to the Mary Heersink School of Global Health”.

**Response: “**May Thanks go to the Mary Heersink School of Global Health and Social Medicine…”

41. Reviewer said on page 27. “Professor Bannerman and Sara”

**Response:** Bannerman Sara
