# Supplementary material for: The Right to Development and disparities in healthcare access: Qualitative study from rural Ontario, Canada
Source: PLOS Glob Public Health. 2026 Jun 1;6(6):e0005758. doi: 10.1371/journal.pgph.0005758 (PMC13225652; doi:10.1371/journal.pgph.0005758)
Supplement: Attachment — Submitted filename: Response _to Reviewers.docx [file pgph.0005758.s004.docx]

**Response to Reviewers**

**Response to reviewers according to line numbers indicated in the revised manuscript with Truck changes**

Reviewers said: a) Please respond to the reviewer's comment about using the word "emerge/emerging" when referring to your thematic analysis. If you agree with their suggestion to refrain from using these terms then please review the paper thoroughly and change this language. If you disagree with the reviewer's suggestion, please provide an explanation in your response letter.

Line 131: The RtD, affirmed by the United Nations General Assembly in 1986, **emerged from** long-standing debates about the indivisibility of civil, political, social, and cultural rights [25,32].

Author response:

The RtD, affirmed by the United Nations General Assembly in 1986, **was shaped by** long-standing debates about the indivisibility of civil, political, social, and cultural rights [25,32].

Line 202: Policy change **emerged i**n the mid-20th century, beginning with Ontario’s first human rights law in186 1944 and expanding through the consolidation of anti-discrimination legislation in 1962.

Author response: Policy change **was initiated** in the mid-20th century, beginning with Ontario’s first human rights law in and expanding through the consolidation of anti-discrimination legislation in 1962.

Line 274 The themes were not predetermined. Instead, they **emerged** directly from the interview data through an iterative process of coding and comparison.

Author response: The themes were not predetermined. Instead, they **derived** directly from the interview data through an iterative process of coding and comparison.

Line 498: responsive healthcare **emerged** as a consistent theme.

Author response: responsive healthcare **was identified** as a key theme.

Line 695: For immigrants, isolation also **emerges** from the loss of familiar networks and environments [76].

Author response: This line deleted.

Line 847-848: This aligns with the “basic needs” approach to development, which **emerged f**rom the recognition that economic growth alone cannot eliminate poverty or secure well-being.

Author response: This aligns with the “basic needs” approach to development, which **developed from the recognition** that economic growth alone cannot eliminate poverty or secure well-being.

Reviewers said:

b) I concur with the reviewer that your discussion often ends up straying from the RtD framework when you reference  philosophers, Said and other theories etc. Please revise this section so that it remains focused on RtD and does not introduce too many other theories.

Reviewers required to change in line 654:-655: Said’s Orientalism illustrates how Western depictions of Arab and Muslim societies justified dominance [63], and these misrepresentations continue to influence policy and public attitudes. Addressing these long-standing boundaries requires more than policy change; it demands engagement with deeper cultural and social divides.

Author response: Within a RtD framework, equitable access to healthcare requires attention not only to material resources but also to the social conditions that shape inclusion and participation. In Oxford County, patterns of social separation and differential treatment reported by participants indicate that racialised boundaries continue to influence healthcare experiences. While existing literature (e.g., discussions of “Self” and “Other” or Orientalism) helps contextualise these dynamics, the RtD framework highlights that such exclusion directly undermines individuals’ ability to participate in and benefit from healthcare systems. Addressing these barriers is therefore essential to fulfilling RtD principles of equity, inclusion, and non-discrimination [63].

Author response in line 693-694: Durkheim’s concept of anomie highlights weakened social bonds [75], while Marx’s notion of alienation points to separation rooted in economic structures. For immigrants, isolation also emerges from the loss of familiar networks and environments [76].

*Change it:* Weakened social ties limit individuals' ability to participate fully in society, a concern central to the RtD framework. This aligns with Émile Durkheim's concept of anomie, which describes the breakdown of social bonds and collective norms [75]*.* Similarly, Karl Marx’s notion of alienation points to a related but distinct form of disconnection one rooted in economic structures, where individuals are separated from the products of their labour, from their own productive activity, and from their human essence. Both anomie and alienation, despite their different origins, identify conditions that undermine the social and economic foundations necessary for meaningful participation in development [76]. These patterns reflect broader processes of social exclusion that can limit participation and access to resources, which are central concerns within the RtD framework.

Line 94: John Rawls' philosophical framework emphasises that everyone should have access to 'basic social goods'. ….

Author changed it: John Rawls' philosophical framework emphasises that everyone should have access to "basic social goods," a principle that aligns with the RtD’s commitment to equitable access to resources and opportunities [24].

Line 157: This limitation highlights a key critique of liberal theories, such as Dworkin’s equality of resources, which protect individuals from bad luck without directly addressing accumulated advantage [37].

Author adds to sentence of: This limitation highlights a key critique of liberal theories, such as Dworkin’s equality of resources, which protect individuals from bad luck without directly addressing accumulated advantage [37]. The RtD addresses this gap by insisting on not only formal equality of opportunity but also substantive measures to redress structural barriers and ensure that historically disadvantaged groups can participate in and benefit from development [31]. In contrast, Sen and Nussbaum's capabilities approach, which focuses on securing substantive freedoms for individuals to lead dignified lives, has informed the RtD's emphasis on participation and equitable benefit-sharing [25].

In line 759-760: Drawing on **Max Weber’s** analysis of social action and authority, cultural characteristics can also shape what [96] calls “monopolistic closure,” a process in which dominant groups use shared traits to secure exclusive access to valued resources, thereby excluding others.

Author changed to: Drawing on **Max Weber’s** analysis of social action and authority, cultural characteristics can also shape what [96] calls “monopolistic closure,” a process in which dominant groups use shared traits to secure exclusive access to valued resources, thereby excluding others. This form of exclusion is precisely what the Rtd framework seeks to dismantle. Cultural responsiveness in healthcare is therefore essential because when systems fail to accommodate diverse backgrounds, they reproduce the very exclusion that the RtD framework seeks to address. The RtD requires that states ensure equal access to essential resources, including healthcare, without discrimination a principle consistent with Article 8 of the 1986 Declaration on the Right to Development, which guarantees equality of opportunity in access to health services and other basic resources which has identified in Art 8 of UN General Assembly [25].
